# Biomechanical assessment of remote and postinfarction scar remodeling following myocardial infarction

**DOI:** 10.1038/s41598-019-53351-7

**Published:** 2019-11-14

**Authors:** Mihaela Rusu, Katrin Hilse, Alexander Schuh, Lukas Martin, Ioana Slabu, Christian Stoppe, Elisa A. Liehn

**Affiliations:** 1 Institute for Molecular Cardiovascular Research (IMCAR), University Hospital, RWTH Aachen, Aachen, Germany; 2 Department of Cardiology Pulmonology, Angiology and Intensive Care, University Hospital, RWTH Aachen, Aachen, Germany; 3 Department of Intensive Care Medicine, University Hospital, RWTH Aachen, Aachen, Germany; 4 Institute of Applied Medical Engineering, Helmholtz Institute, Medical Faculty, RWTH Aachen University, Aachen, Germany; 5 Human Genetic Laboratory, University of Medicine and Pharmacy Craiova, Craiova, Romania

**Keywords:** Cardiovascular biology, Cardiology, Nanoscale biophysics

## Abstract

The importance of collagen remodeling following myocardial infarction (MI) is extensively investigated, but little is known on the biomechanical impact of fibrillar collagen on left ventricle post-MI. We aim to identify the significant effects of the biomechanics of types I, III, and V collagen on physio-pathological changes of murine hearts leading to heart failure. Immediately post-MI, heart reduces its function (EF = 40.94 ± 2.12%) while sarcomeres’ dimensions are unchanged. Strikingly, as determined by immunohistochemistry staining, type V collagen fraction significantly grows in remote and scar for sustaining de novo-types I and III collagen fibers’ assembly while hindering their enzymatic degradation. Thereafter, the compensatory heart function (EF = 63.04 ± 3.16%) associates with steady development of types I and III collagen in a stiff remote (12.79 ± 1.09 MPa) and scar (22.40 ± 1.08 MPa). In remote, the soft de novo-type III collagen uncoils preventing further expansion of elongated sarcomeres (2.7 ± 0.3 mm). Once the compensatory mechanisms are surpassed, the increased turnover of stiff type I collagen (>50%) lead to a pseudo-stable biomechanical regime of the heart (≅9 MPa) with reduced EF (50.55 ± 3.25%). These end-characteristics represent the common scenario evidenced in patients suffering from heart failure after MI. Our pre-clinical data advances the understanding of the cause of heart failure induced in patients with extended MI.

## Introduction

Myocardial infarction (MI) is the most prevalent pathology of cardiovascular diseases causing increased mortality and morbidity worldwide^1^. Survivors of MI undergo a substantial risk of developing heart failure through a dynamic remodeling process of the ventricles. In response to ventricle remodelling after MI, scar tissue replaces the infarcted heart area. Cardiac remodeling includes morphological modifications of ventricle chamber by which cardiac function is impaired^2,3^. These modifications include changes of the cavity diameter, mass, ventricle wall thickness and shape, area size of the scar and its spreading after MI, and collagen matrix remodeling. The dilatation of ventricle is associated with myocyte loss, resulting from the chronical myocardial ischemia. Depending on the duration and the magnitude of myocardial stress, the surviving myocytes preserve their contractility function by increasing the number of sarcomeres (hypertrophy), while the interstitial collagen changes its composition and morphology^4,5^. The abnormal accumulation of the collagen^6,7^ and the reduction of scar compliance^8^ diminishes the myocytes’ contractility in the remote area, while the overall heart reduces its compliance and diastolic function. This reduction may eventually lead to heart failure. On the one hand, in the scar the association of ventricle stiffness with collagen accumulation is under debate^6,7^, as the remodeling processes not always reduce the heart function^9–11^. On the other hand, in remote, the contraction of the cardiac muscle obeys Frank-Starling law^12–17^ and sarcomeres stretch to optimum myofilaments overlap in the attempt to counterbalance the biomechanical turnover of scar and remote, thus preserving the pump function of heart^18–22^. Adaptive conditions of the infarcted heart to compensate for pressure overloads results in an abnormal accumulation of fibrilar collagen in fibrotic scar^19^. In the heart, type I collagen is the major fibrillar component (normally > 50% of newly synthesized collagen), hence considered as the mechanical key player for controlling the deformation and myocardium rupture^23^. In a normal heart, type III collagen is of about 10–45% of total collagen^23^. Timely, 2- and 4-days after myocardial infarction, biosynthesis of types I and III collagen normalizes the developed high pressure in the infarcted region of heart rats^24^. Type I collagen is a relative stiff protein with a pronounced non-linear stress-strain dependence due to its adaptability to uncoil upon elongation^25^. Type I collagen presumably opposes sarcomeres overstretching and determines tissue stiffness. In contrast, type III collagen fibers have a lower tensile strength than that of type I collagen fibers, and presumably are more suitable to maintain structural integrity of collagen network^26^.

Other collagen subtypes are yet not studied in relation to cardiac fibrosis after MI. However, we found that type V collagen, although at minimal amounts in the heart (<5%^27^), may play a critical role in collagen fibers formation^28^. The Col5a1 knock out mice have a constitutional cardiovascular insufficiency, since type V collagen presence is essential for stabilizing the injured tissue^28^. *In vitro*, type V collagen has a central role in early co-assembling of *de novo* heterotypic structures of types I and III collagen molecules^28,29^. Additionally, type V collagen coated cultures vs. s types I and III coated cultures seems to be more resistant against an early enzymatic degradation of *de novo* collagen fibers^30^.

However, the functional consequences of regional alterations of amounts of fibrilar type I, III, and V collagen on diastolic and systolic heart function and tissue biomechanics have yet only been partly investigated. Higher expression of type I collagen vs. type III collagen seems to associate with a slower relaxation rates of left ventricle^31^, whereas an early increase of type III collagen expression corresponds to a decrease of myocardial stiffness^27^. Although an increase of total collagen expression associates with increased left ventricle stiffness^32^, no functional consequence can be drawn regarding the causative effect of fibrillar collagen subtypes during the course of myocardial remodeling^9–11^. The evidence of collagen functionality on physiological and morphological characteristics of the heart may improve the treatment strategies in the context of MI. Mechanical models based on size and stiffness modifications reveal that large and compliant infarcts lead to lower cardiac performance than more rigid ones^33,34^. The anisotropically oriented collagen fibers in the infarcted region^35^ are responsible for preserving the ejection fraction in the post-MI process by increasing diastolic filling and myocytes contractility through the Franklin-Starling mechanism^12,13^.

However, a multidisciplinary approach can bring additional knowledge for understanding the interdepending dynamical remodeling processes of remote and infarction scar. Correlative models relating the spatial distribution of collagen composition post-MI and the effect of biomechanical characteristics of collagen fibers on regional tissue remodelling are yet to be envisaged. Based on this background, the present study focuses for the first time on the causative role of biomechanical characteristics of types I, III, and V collagen on patho-physiological conditions of the heart within the healing processes after MI. Therefore, we do anticipate that our scientific report will contribute to delineating the critical molecular and biomechanical regulators of infarcted heart undergoing temporal remodelling; hence, bringing valuable knowledge and expanding the state of the art on tissue formation and remodeling such as remote and scar in other fields.

## Methods

### Ethical statement

All animal experiments and study protocols were performed at the University Hospital of the RWTH Aachen and approved by local authorities, complying with German animal protection laws and performed according to the guidelines from Directive 2010/63/EU of the European Parliament (ethics approval granted by Landesamt für Natur- und Verbaucherschutz (LANUV) Nordrhein-Westfalen (NRW) Nrs. 8.87-50.10.35.09.088).

### Murine model of myocardial infarction

All experiments are conducted conform to the current practical guidelines for rigor and reproducibility in preclinical studies^36,37^. All operated animals were included in the study, unless they died during the surgery or the tissue samples were damaged during the histological preparation. For statistical analysis one-way ANOVA and Newman-Keuls post-test was used. The estimates of standard error and the accurate p value was reported. Eight-week-old male C57Bl/6J mice (Charles Rivers, Cologne, Germany), underwent an open-chest myocardial infarction (MI) as previously described^38^. In brief, mice were initially anesthetized by intraperitoneal injection of 100 mg/kg ketamine and 10 mg/kg xylazine (Medistar, Ascheberg, Germany) and intubated with positive pressure ventilation with oxygen and isofuran 1% (Abbott, Hannover, Germany) by using a rodent respirator. Subsequently, hearts were exposed by left thoracotomy and MI was produced by left anterior descending artery (LAD) permanent ligation. The muscle layer and skin incision were closed with a silk suture. Animals (n = 42) were divided into 7 groups (n = 6/group) and sacrificed before and at 1, 4, 7, 14, 21, and 28 days post-MI. The existent data and the existent tissue samples were randomly used and data analysis were blindly performed by scientists.

### *In vivo* functional ultrasound imaging

The function of mice hearts (n = 5 per each time point) was investigated *in vivo* by functional ultrasound imaging at different time points (before and 1, 4, 7, 14, 21, and 28 days post-MI). Two-dimensional B-mode and M-mode echocardiographic measurements (Vevo All770, Visual Sonics, Toronto, Canada) were performed using a 40 MHz transducer having the mouse placed under anaesthesia with 1.5% isoflurane, in supine position on a warming pad (Vevo Mouse Handling Table) for the whole imaging time. Images of the cardiac long and short axis were recorded for quantifying the left ventricular cavity dimensions. Left ventricle ejection fraction (EF %) and fraction shortening (FS %) were estimated during the cardiac cycle by means of Vevo 3.1.0. software and reported as percentage. M-Mode of long axis (before papillary muscles) was used to measure the wall thickness. All primary measurements were manually traced by a blind trained scientist. The computed data were fitted by Boltzmann equation using OriginPro 2017 (ADDITIVE, Germany) and statistically represented (±SEM). The condition of the heart pump function will be addressed according to the classic classification, such as normal (compensatory) for EF > 60%, mild for EF > 50%, and reduced as EF ≤50%^39^.

### Tissue section preparation

Mice heart (n = 4–5 per group) were excised according to the groups studied at various time points after MI, fixed in formalin (Carl Roth, Karlsruhe, Germany), and embedded in paraffin (Vogel Histo-Camp, Germany). Serial tissue sections (5 μm) of 10–12 sections per heart, 400 µm apart, up to the mitral valve were cut. Tissue sections were deparaffinated in xylene, rehydrated with a graded series of ethanol and subjected further to histochemistry, immunohistology, and biomechanics for the quantification of total collagen, types I, III, and V collagen, and tissue stiffness, in remote and scar regions. Remote tissue is defined as the viable area of the ischemic heart, whereas scar area is the fibrotic tissue which replaced the normal tissue after infarction^40^.

### Histochemical staining of total collagen

For histochemical analysis of collagen, the Gommori’s trichrome method was used^41^. In short, deparaffinated heart tissue sections (n = 10/mouse/time point) were fixated with Bouin’ solution (Sigma-Aldrich, Darmstadt, Germay), followed by Weigert’s iron haematoxylin (Sigma-Aldrich, Darmstadt, Germay) staining and Gomori’s trichrome staining solution. Histological images of 2 sections per mouse and 9 images per section were recorded (40x objective) with Diskus software (Hilgers, Königswinter, Germany) by using Leica Microscope DM 2500 (Leica, Amsterdam, Netherlands) and analyzed by means of commercially available ImageJ software (National Institute of Mental Health, Bethesda, Maryland, USA). Using threshold area fraction determination, the percentage of collagen positive areas was calculated. The amount of collagen was reported as a percentage from the total number of pixels in the optical view as percentage and expressed as mean ± SEM^40^. The analysis was blindly performed by two scientists.

### Immunohistochemistry staining of types I, III, and V fibrillar collagen

Two sections per mouse and 9 images per section were recorded (40x objective) in six different fields per section in remote and scar Leica Microscope DM 2500. The amount of types I (rabbit antimouse type I collagen 1:100, Cedarlane, Burlington, Canada), III (rabbit antimouse type III collagen 1:100, Abcam, Cambridge, Great Britain), and V (rabbit anti-mouse type V collagen 1:100, Abcam, Cambridge, Great Britain) collagen was analyed using color threshold area fraction determination in ImageJ software. The positive-stained area of each subtype of collagen was normalized to the total fibrillar collagen (remote and scar areas), and expressed as mean ± SEM. Contour plots for each type of collagen in remote and scar tissue are produced with the algorithm delivered with the software OriginPro 8G (ADDITIVE, Germany) by triangulation and linear interpolation of the measurement values. The contour plots display parameter fields of EF and stiffness with similar collagen fractions with the same colour. For selected collagen fractions, isolines are drawn.

### Time dependent of periodical structures of sarcomeres

Deparaffinized, unstained, tissue specimens were transferred to the liquid-BioScope atomic force microscope (AFM) instrument (Bruker, Santa Barbara, USA). The AFM images of two sections per mouse and 3 images per section were recorded in ddH_2_O using a silicon nitride probe with the calibrated spring constant of 0.2 N/m and a defined tip radius of 8 nm. The scan rate and pixels of 0.2 Hz and 512, respectively were chosen for detailed observations. Surface tracking, and hence image quality was optimized by lowering the scan rate and scanning along myocardial cell direction. A lowpass Gaussian filter on both X and Y directions was applied and led to an increased signal-to-noise ratio, which ensured the increased details of features at the meso-scale. The composition and structure of heart tissue might be altered by sample preparation. For example, the formalin fixed paraffin embedding process might shrink tissues and induce significant loss of protein phosphorylation, thereby inducing possible modifications at the nano-scale. However, we assumed that these effects are equally distributed in all group samples and thereby the relative comparison of the results can be considered of significant relevance.

### Force measurement

The regional stiffness of the heart tissue and sarcomeres was performed by using liquid-atomic force microscopy-BioScope, Bruker, Santa Barbara, USA. PeakForce quantitative nanomechanics method was used as the unique method to analyse mechanical properties of heart tissue samples and to correlate them to tissue remodeling. The BioScope integrated fluorescence light microscope and the microscope image registration and overlay software guided the tip and measurements into a selected region of interest without compromising the performance of AFM measurements. The X-Y AFM stage of ≥150 μm allowed scanning of large images for the statistical determination of sample stiffness. All peak-force measurements were continuously recorded in demineralized water while raster scanning. This method enables the acquisition and analysis of individual force -distance curves from each tap recorded during tissue imaging. Under very small forces between tip and sample surface, once the tip is brought to the proximity of the sample surface the tip deflects. The tip deflection is a measure of tissue stiffness. Stiffness and topology maps were recorded simultaneously using calibrated micrometre-size hydra-V-shape probes with a nominal spring constant of 0.2–0.5 N/m with cantilever length 200 μm. All force measurements were conducted in ultrapure water, at room temperature. The absolute unit of Young’s modulus (MPa) was obtained statistically from an average of 50 force-distance curves per image and 256 data points per force curves. The noncontact and artefacts regions were excluded from Young’s modulus calculation. Each force curve was converted to a force separation plot and further analysed by using of Derjaguin, Muller, Toropov (DMT) model^42^ (Eq. 1) to determine the Young’s modulus values.

1 $${F}_{tip}=\frac{4}{3}{E}^{\ast }\sqrt{R{d}^{3}}+{F}_{adh}$$

where *F* is the force, *R* is the tip radius, *d* is the separation distance, *F*_*adh*_ is the adhesion force, and E^*^ the reduced modulus. The reduced modulus (elastic deformation of both sample and probe) was obtained by fitting the linear part of the retract curve. Provided that infinite elastic modulus for the tip (*E*_*tip*_) applies and knowing that the Poisson’s ratio of the sample, ν_sample_ is 0.5, the Young’s modulus for the tissue samples was estimated based on the Eq. 2.

2 $${E}^{\ast }=(\frac{1-{\nu }_{sample}^{2}}{{E}_{sample}}+\frac{1-{\nu }_{tip}^{2}}{{E}_{tip}})$$

The deflection sensitivity was calibrated against a clean glass slide in air by repeated contact mode indentation. The AFM cantilever spring constant was determined to be *k* = 0.22~0.25 Nm^−1^ by using the Thermal Tune method^43^. Each tissue region and sarcomeres were qualified as soft or hard relative to the tissue stiffness values of tissues or sarcomeres of Wilde Type mice (control).

### Statistical analysis

Data were reported as the average ± SEM for the number of samples reported for each method, unless otherwise stated. Statistically significant differences of each data set evaluated in remote and scar at every time point after MI were calculated vs. control by means of one-way ANOVA and Newman-Keuls post-test. A value of P < 0.05 was considered significant.

## Results

### *In vivo* functional ultrasound imaging

**Figure 1:**
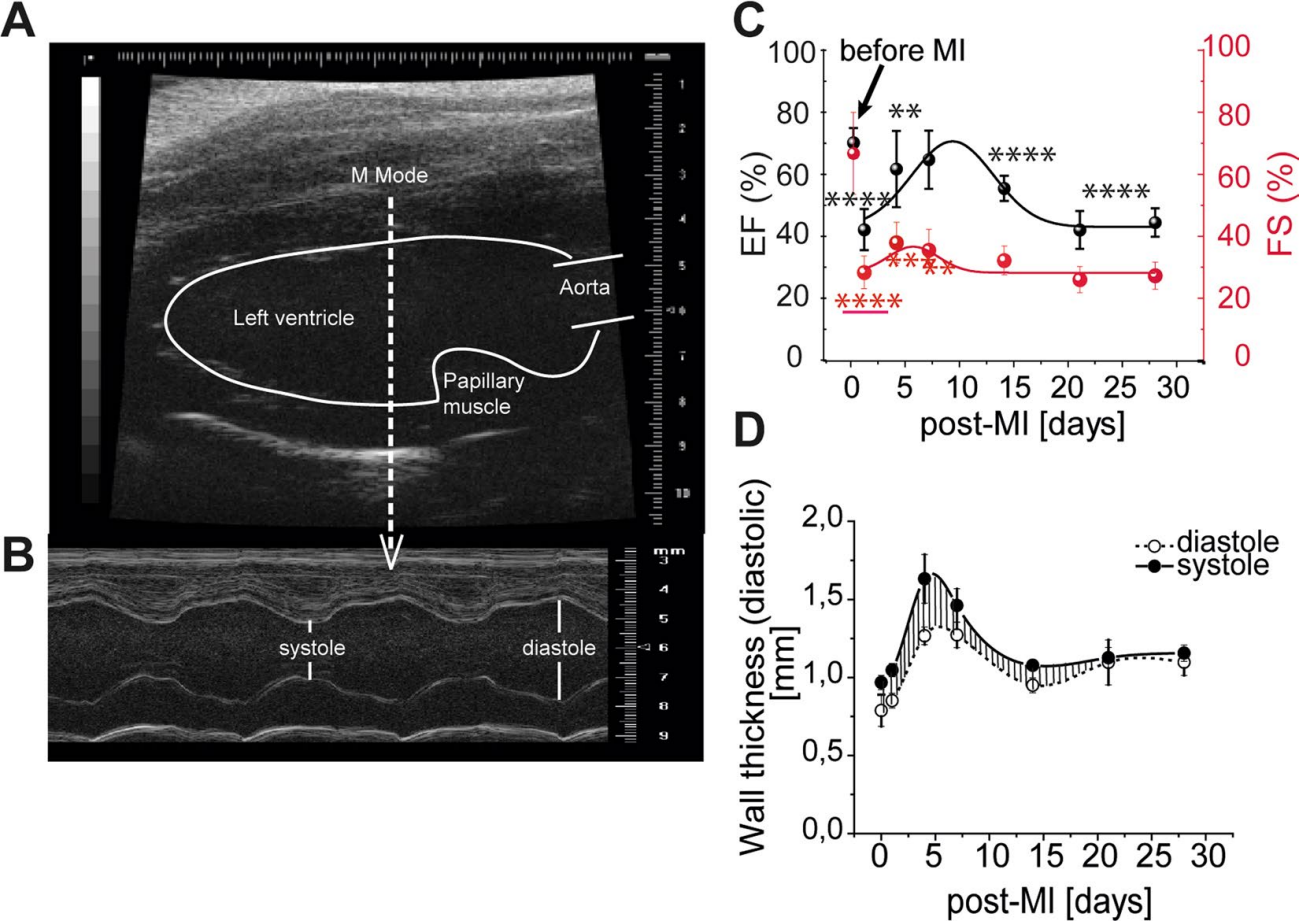
Representative two-dimensional murine cardiac echocardiography measurements. (**A**) Long axis B-mode image showing the tip of the left ventricle cavity, the papillary muscle, and the origin of the aorta. The cursor placed in front of the papillary muscle, towards the apex, indicates the exact position where the M-mode images were recorded. (**B**) Long axis M-mode image showing the left ventricle movement during the cardiac cycle and an example of left ventricle -cavity and -walls measurements (left side of the image). (**C**) Left ventricular activity fraction, EF [%] (black line, left y axis) and FS [%] (red line, right y axis) during the healing post-MI period of 28 days, (**D**) Left ventricle wall thickness measured in M-Mode at different time points after MI; closed circle: systole, open circle: diastole (**p < 0.01, ***p < 0.001, ****p < 0.0001).

The heart function of mice (n = 6/time point) at different time points after MI was investigated by functional ultrasound imaging. The 2D echocardiographic images of the cardiac long axis (Fig. 1A) were used to estimate the ejection fraction (EF %) and fraction shortening (FS %). The M-mode was used for determining the wall thickness (Fig. 1B). The time - dependent EF (%) and FS (%) were fitted with a b-spline fitting function to determine the maxima of EF and FS, respectively (Fig. 1C). The mean EF % and FS % significantly decreased at day 1 post-MI corresponding to a reduced heart function (from 70.1 ± 4.8% to 42.1 ± 6.6% (p < 0.0001) and from 66.7 ± 13.2% to 28.3 ± 5.3% (p < 0.0001), respectively). EF % and FS % recovered temporarily at 9- and 6 days post-MI, to 70.1 ± 4.8% and 37.9 ± 6.5%, respectively. Thereafter, it decreased again at later time points at the values comparable with those directly after MI. The left ventricle wall thickness varies in time, from 0.2 ± 0.05 mm to a maximum of 0.4 ± 0.10 mm at 4 days post-MI, probably due to the interstitial oedema. Thereafter, they decreased at 14 days post-MI to 0.13 ± 0.01 mm (Fig. 1D). Remarkably, although the compensatory mechanism seemed to re-establish the heart function during healing after MI, without any therapeutical intervention the end- outcome progressed towards a heart failure.

### Total collagen in remote vs. scar

**Figure 2:**
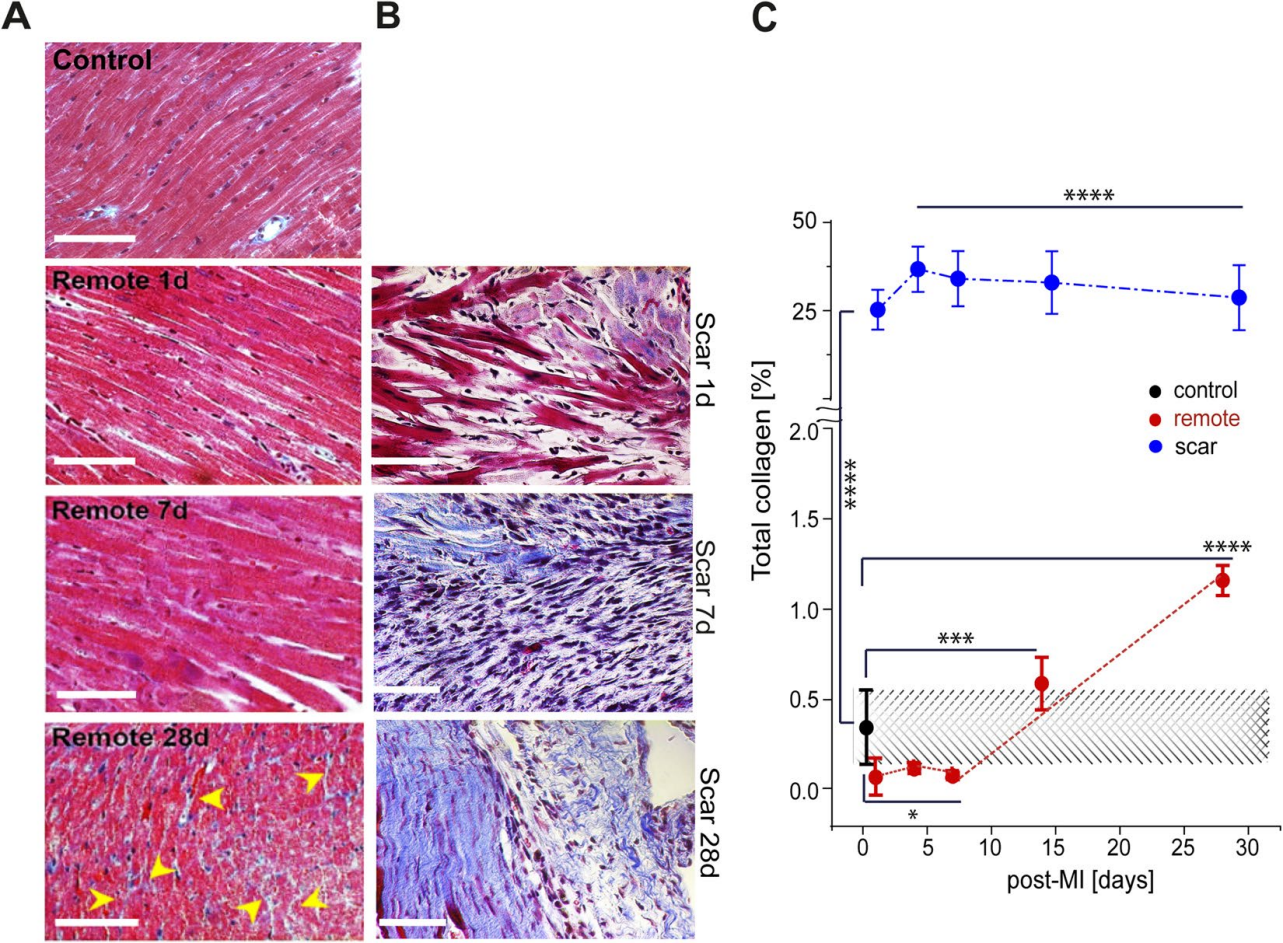
Total collagen distribution and turnover in infarct scar vs. remote tissue within the healing period up to 28 days post-MI. (**A**) Gomori’s Trichrome staining images of control and remote vs. (**B**) scar. Scale bar: 50 μm. (**C**) Time dependent measurements of total collagen amount in remote and control vs. scar within 28 days post-MI. The red and blue lines are guides to the eye. Collagen accumulates significantly in the infarct scar vs. remote and control. After 7 days post-MI, collagen increases linearly in remote and decreases linearly in the infract scar. (*p < 0.5, ***p < 0.001, ****p < 0.0001).

The expression and spatial distribution of total collagen was evaluated by histological Gomori’s trichrome staining in both controls (no surgery) and murine-mice model (n = 4–5 per group). Total collagen accumulated gradually by 28 days post-MI in both remote (Fig. 2A) and scar areas (Fig. 2B). In the scar, the mean collagen content at day 1 and 4 post-MI increased significantly (25.2 ± 1.51%; 36.70 ± 1.66%, respectively, p < 0.0001) compared to that of control. Thereafter, by 28 days post-MI it decreased to 28.65 ± 2.30% (p < 0.0001). In remote areas, the quantification of total collagen showed that after an initial decrease at 7 days post-MI from 0.3 ± 0.1% to 0.1 ± 0.01% (p < 0.05), the total collagen content also increased by 28 days post-MI to a maximum of 1.2 ± 0.8% (p < 0.0001) (Fig. 2C). Side-by-side collagen quantification evidenced that, the collagen matrix remodelling is not only a characteristic of the scar, but of the entire heart, including the viable regions, such as remote.

### Expression of types I, III, and V collagen in remote vs. scar tissues

**Figure 3:**
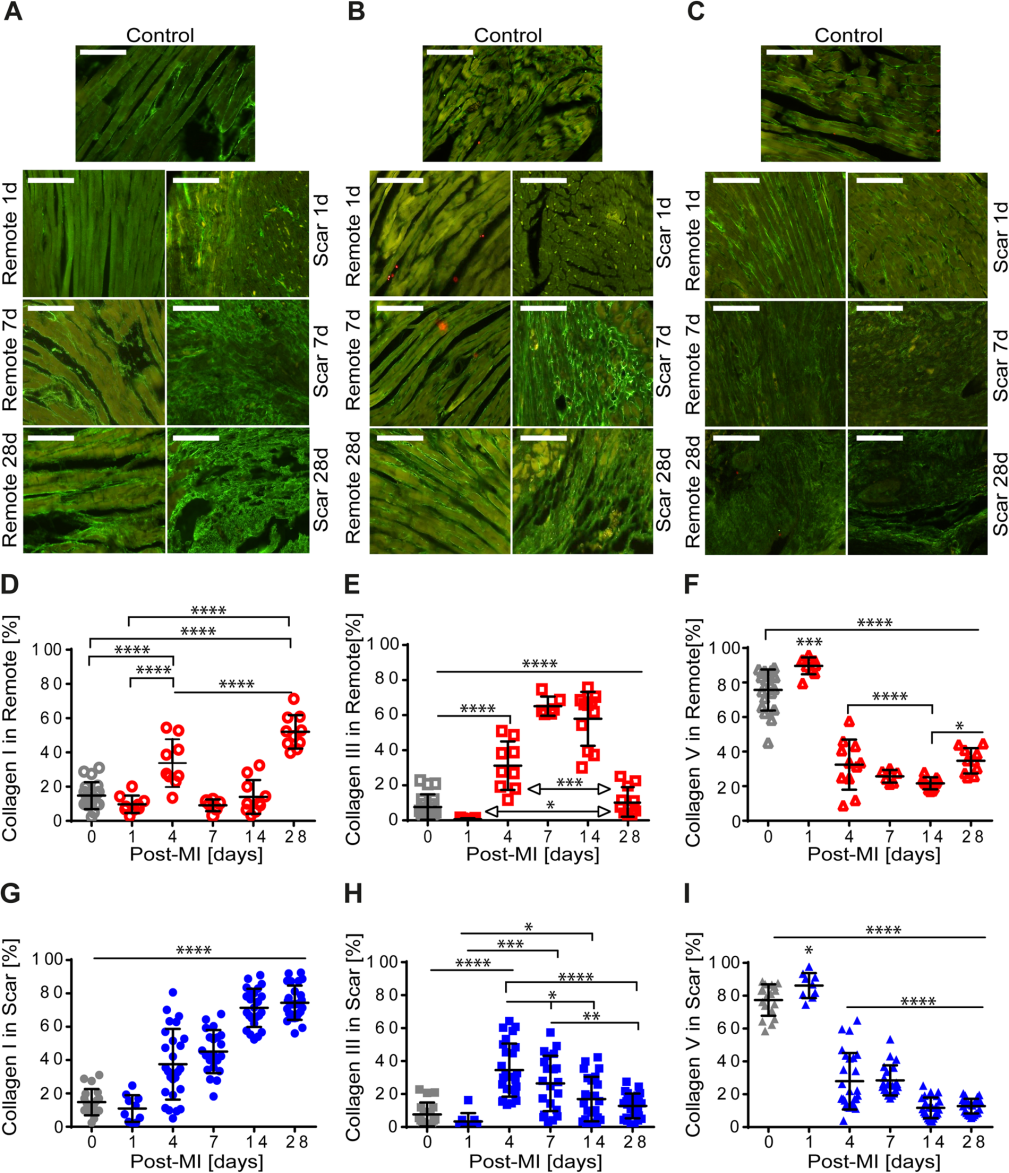
Immunohistological characteristics of types I, III, and V collagen in control and remote vs. scar within the healing period of 28 days post-MI. Spatial distribution of type I collagen in remote (**A**-left) and scar **(A**-right), type III collagen in remote (**B**-left) and scar **(B**-right), and type V collagen in remote (**C**-left) and scar **(C**-right) control (upper panels). Scale bar: 50 μm. Time variation of the amount of types I, III, and V collagen in remote (**D–F)** and in scar **(G–I**). The collagen amount is normalized by the total amount of type I, III, and V collagen in remote and scar. (*p < 0.5, ***p < 0.001, ****p < 0.0001).

The spatial distribution and quantification of types I, III, and V collagen was evaluated by means of immunohistological methods (n = 4–5 per group). Timely, the staining intensity of both types I and III collagen were more visible in the scar (Fig. 3A,B -right) than that in remote (Fig. 3A,B -left). Staining intensities of type V collagen were elevated at 1-day post-MI and seemed to decline by 28 days post-MI in both remote (Fig. 3C -left) and scar (Fig. 3C -right) regions.

The quantification of type I collagen showed its increase in remote with an alternating pattern with two maxima of 33.8 ± 4.65% and 52.0 ± 3.10% at 4 days and 28 days post-MI, respectively (Fig. 3D). In the scar, type I collagen gradually increased to a steady plateau value (71.3 ± 2.34%) at 14 days post-MI (Fig. 3G). By day 28 post-MI, in the scar type I collagen increased about 1.5-fold more than that of control mice and remote regions (14.8 ± 1.81%; p < 0.0001) (Fig. 3D,G).

In remote, type III collagen expression showed a maximum at 7 days post-MI (65.2 ± 2.25%) (Fig. 3E) while it decreased by a factor of approximately 5 (13.3 ± 3.16%; p < 0.0001) by 28 days post-MI compared to that of type I collagen (Fig. 3G). The amount of type III collagen in the scar, peaked at 4 days post-MI (34.6 ± 3.16%) (Fig. 3H) and at 28 days post-MI it decreased by a factor of about 3 (12.8 ± 1.5% (p < 0.0001)).

The expression of collagen V significantly increased in both scar and remote regions at one day post-MI (remote: 89.7 ± 1.63% vs. scar: 86.2 ± 2.54%; p <0.001) (Fig. 3F,I). Independent of its spatial distribution, at one day post-MI, these values were one-, two- and three-order of magnitude higher than those of types I and III collagen, respectively (Fig. 3D,E,G,H). Timely, the decay of type V collagen expression follows different decays in remote and scar, specifically. In remote, its expression decreased continuously by 14 days post-MI (21.7 ± 1.04%) (Fig. 3F). However, in the scar region, type V collagen decayed abruptly at 4 post-MI (27.94 ± 3.37%, p < 0.0001) and at 14 post-MI (12.76 ± 0.89%, p < 0.0001) (Fig. 3I). By 28 days post-MI, type V collagen fibrils remained at low abundance in both remote and scar regions. Taken together, we showed that types I, III, and V collagen fractions accumulated simultaneously but with different proportions. Their expression patterns were regionally and timely dependent.

### Sarcomeres dimensions and stiffness variation under MI conditions

**Figure 4:**
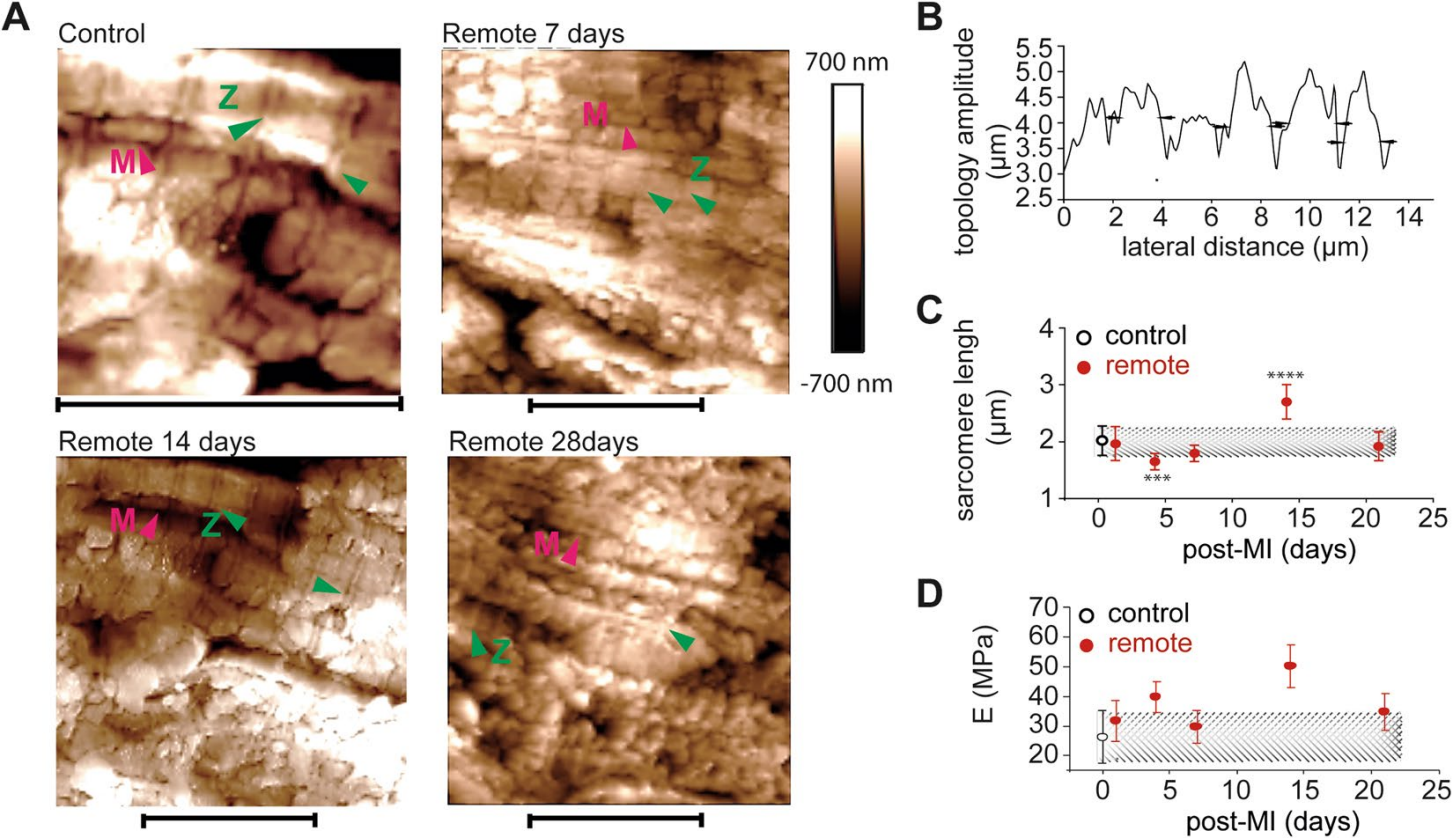
Odd and even dyssynchronous contraction of sarcomeres in remote tissue vs. control within 21 days post-MI. (**A**) High-resolution AFM image topology of sarcomeres in control and remote areas (20 topological lines per image with n = 256 samples/image line) separated by transversal structures of Z bands (green head arrows). The globule-like subsurface structures displayed in between sarcomeres indicate mitochondria (M, red head arrows). Scale bar: 10 μm x 10 μm for control and 20 μm x 20 μm for remote area, respectively. (**B**) Typical example of topology of sarcomeres in control tissue reveals structures with a periodicity of (2.0 ± 0.7) μm. (**C**) Time dependent variation of sarcomere length (δ) in remote vs. control. Comparisons for statistical significance at all time points were performed relative to control values. (**D**) Time-dependent stiffness variation of sarcomeres in remote vs. control tissues (***p < 0.001, ****p < 0.0001).

High-resolution AFM was used to illustrate and determine the extent of sarcomere elongation (δ) within healing post-MI. The parallel longitudinal contractile units with corresponding transversed Z and M bands structures (sarcomeres) (Fig. 4A) were visible in height AFM images as well-defined periodical structures (Fig. 4B). Globular features displayed between sarcomeres assigned potentially the subsurface mitochondria. In case of the control tissue, the mean sarcomere’s length (δ_control_) was 2.0 ± 0.3 μm (Fig. 4C), which was similar to that of remote at 1-day post-MI. The length of sarcomere decreased significantly at 4 days post-MI (1.6 ± 0.03 μm) followed by a gradual increase (1.33-fold; p < 0.0001) at 14 days post-MI. By 21 days post-MI, δ_remote_ returned to its similar level at first day post-MI (1.9 ± 0.2 μm; p < 0.0001). The sarcomeres’ length seemed to be time-dependent influenced. It might be involved in temporally compensation of EF% regime after MI. Force distance curves were recorded simultaneously with topology images and further used to calculate sarcomeres stiffness (Fig. 4D). The mean stiffness of sarcomeres followed an odd and even effect within 28 days post-MI. The mean sarcomere stiffness increased significantly at 4 days post-MI (39.8 ± 3.8 MPa, p <  0.01) and 14 days post-MI (50.1 ± 3.7 MPa; p < 0.0001) compared to that of control (26.3 ± 1.6 MPa). The sarcomeres’ stiffness showed 2 minima values of 29.63 ± 1.9 MPa at 7 days post-MI and of 34.6 ± 2.3 MPa at 21 days post-MI, respectively.

### Local compliance of scar and remote zones

**Figure 5:**
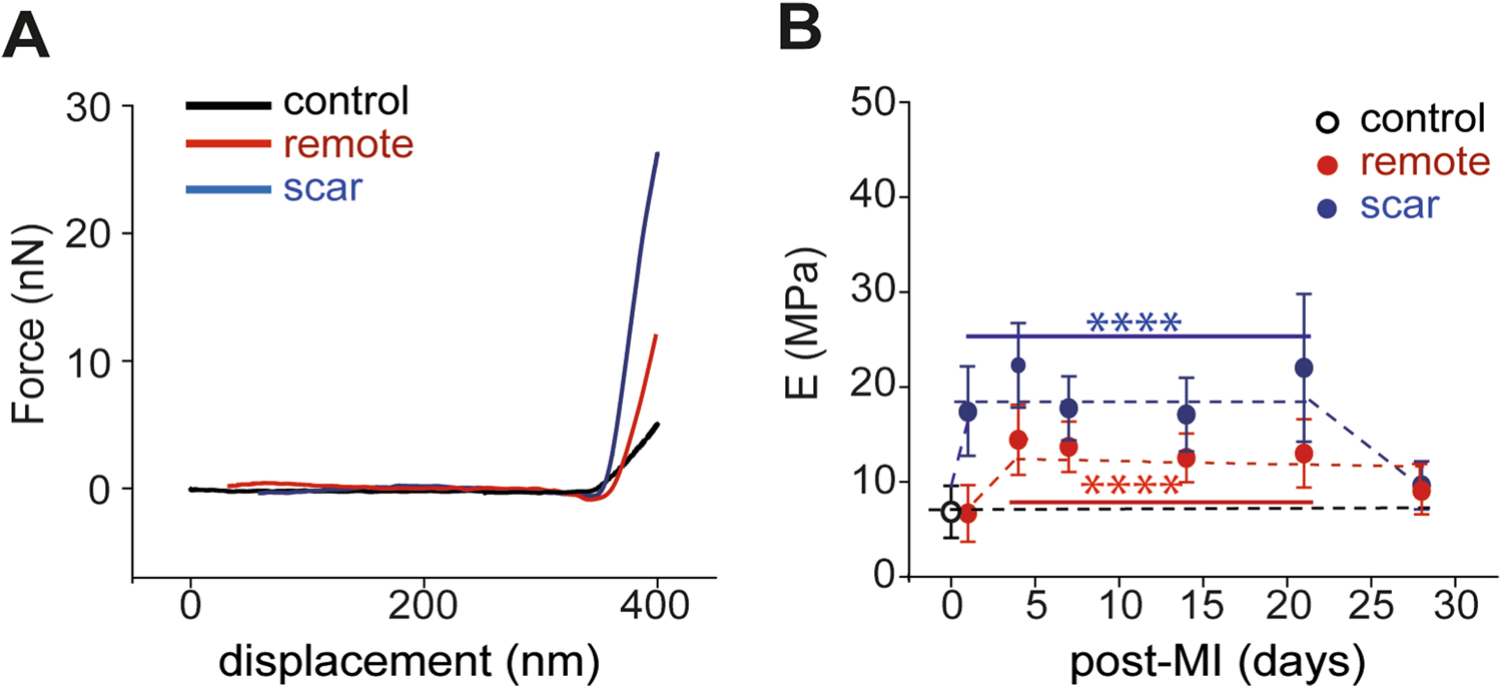
Bio-mechanical characteristics of control (open circle) vs. remote and scar regions. (filled circle) (**A**) Representative force (F) vs. displacement (d) retraction curves recorded on control, scar and remote regions of mice heart tissue. The differences in the slopes of F-d curves indicate different elastic properties (stiffness) of the heart tissue. (**B**) Time dependent biomechanics modification of the E-Modulus (E) in control and remote vs. infarct scar. In the inflammatory phase (7–21 days post-MI) infarct scar undergoes a maximum tension compared to the remote region and it returns at its initial values at 28 days post-MI (****p < 0.0001).

Biomechanical properties of remote and scar were determined by means of AFM. Stepper slope was obtained in case of scar > remote > control tissue that directly associates with the degree of tissue stiffness (Fig. 5A). At day one and day 4 post-MI, both E_scar_ (17.4 ± 4.6 MPa (p < 0.0001)) and E_remote_ (6.9 ± 0.8 MPa (p < 0.0001)) increased significantly to constant values vs. E_control_ = 6.8 ± 2.7 MPa (Fig. 5B). At day 28 post-MI, simultaneously E_remote_ (8.8 ± 1.2 MPa (p < 0.0001)) and E_scar_ (9.6 ± 0.4 MPa (p < 0.0001)) dropped significantly (p < 0.0001) approaching the E_control_ value. Taking together, the biomechanical characteristics varied in a time and regional dependent manner until they reached a stable state.

### Contour plots of types I, III, and III collagen vs. EF% vs. E in remote and scar tissue

**Figure 6:**
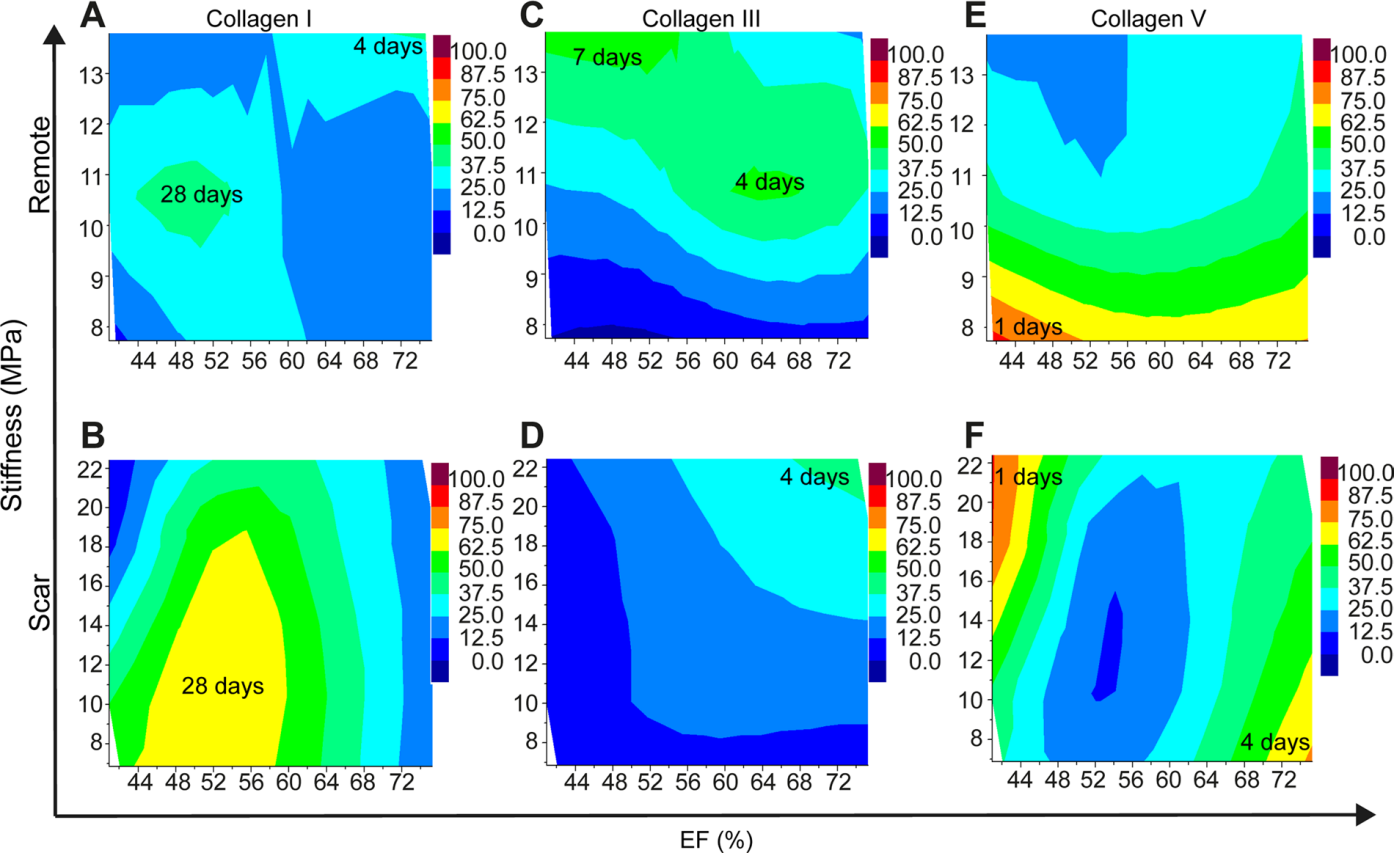
Contour plots displaying the fraction of I, III, and V collagen for different parameter fields of EF (%) and tissue stiffness. Contour plots of the expression of type I collagen in remote (**A**) and scar (**B**), of the expression of type III collagen in remote (**C**) and scar (**D**), and of the expression of type V collagen in remote (**E**) and scar (**F**). Hue color gradients assign the minimum and maximum expression values for type I collagen (**A,B**), type III collagen (**C,D**) and type V collagen (**E,F**) respectively in remote and scar regions. The inset values represent post-MI days that indicate the maximum expression values for each collagen type.

Contour plots displaying parameter fields of EF and stiffness with similar collagen fractions with the same colour are depicted in Fig. 6A–F. Time dependent variation of EF, stiffness, and the expressions of types I, III, and V collagen determined in remote and scar is tabulated in Table 1 and Table 2, respectively. For selected collagen fractions, isolines are drawn. In remote tissue, the highest fraction of type I collagen is visible at medium values of stiffness with temporary compensatory EF (Fig. 6A). In the scar region, the strongest development of type I collagen is seen for low to medium tissue stiffness and EF (Fig. 6B).

**Table 1:** Variation of EF, stiffness, and types I, III, and V collagen expression in remote within 28 days post-MI.

Clinical performance*	Post-MI (days)	EF (%) ± SD	E (MPa) ± SD	Coll I (%) ± SD	Coll III (%) ± SD	Coll V (%) ± SD	Ratio I: V	Ratio III: V	Ratio I: III
Normal	0	75.26 ± 1.75	6.72 ± 0.56	14.78 ± 1.81	7.64 ± 1.71	75.69 ± 2.72	1:2	1:5	2:1
Reduced	1	40.94 ± 2.12	6.87 ± 0.75	9.71 ± 0.64	0.64 ± 0.18	89.65 ± 1.63	1:10	1:100	10:1
Normal	4	63.04 ± 3.16	12.79 ± 1.09	33.77 ± 4.65	31.20 ± 4.60	32.45 ± 4.37	1:1	1.1	1:1
Normal	7	62.49 ± 4.73	10.68 ± 0.39	9.12 ± 1.39	65.15 ± 2.25	25.73 ± 1.50	1:2	2.5:1	1:6.5
Mild	14	52.94 ± 2.47	12. 33 ± 0.64	14. 04 ± 3.12	57.94 ± 4.64	21.66 ± 1.04	1:1.5	2.5:1	1:4
Mild	28	50.55 ± 3.25	8.82 ± 1.61	52. 01 ± 3.1	13.28 ± 3.16	34.71 ± 2.32	1.5:1	1:2.5	4:1

The clinical performance of heart at different time points after MI is done according to^*^ ^39^.

**Table 2:** Variation of EF, stiffness, and types I, III, and V collagen expression in scar within 28 days post-MI.

Clinical performance^*^	Post-MI (days)	EF (%) ± SD	E (MPa) ± SD	Coll I (%) ± SD	Coll III (%) ± SD	Coll V (%) ± SD	Ratio I: V	Ratio III: V	Ratio I: III
Normal	0	75.26 ± 1.75	6.87 ± 0.56	14.78 ± 1.81	7.64 ± 1.71	77.38 ± 2.25	1:5	1:11	2:1
Reduced	1	40.94 ± 2.12	17.21 ± 1.29	10.95 ± 2.53	3.37 ± 1.69	86.18 ± 2.54	1:8.5	1:28	1:3
Normal	4	63.04 ± 3.16	22.40 ± 1.08	37.51 ± 4.16	34.56 ± 3.16	27.94 ± 3.37	1.2:1	1.2:1	1:1
Normal	7	62.49 ± 4.73	17.29 ± 0.66	45.12 ± 2.82	26.43 ± 3.66	28.45 ± 2.01	1.5:1	1:1	1.5:1
Mild	14	52.94 ± 2.47	17.37 ± 1.19	71.34 ± 2.34	16.97 ± 2.75	11.69 ± 1.27	7:1	1:1	4:1
Mild	28	50.55 ± 3.25	9.59 ± 0.39	74.41 ± 2.06	12.83 ± 1.50	12.76 ± 0.89	6:1	1:1	1:1

*The clinical performance of heart at different time points after MI is done according to^39^.

The increased fraction of type III collagen in remote tissue can be associated with the increased stiffness and remains at high level for all EF values (Fig. 6C). In the scar, low fraction values of type III collagen are observed, which increase with stiffness and EF (Fig. 6D).

The highest fraction of type V collagen is achieved for reduced EF and low stiffness (Fig. 6E). In the scar, increased fraction of type V collagen can be associated with increased tissue stiffness and reduced EF as well as decreased stiffness and increased EF (Fig. 6F). Taken together, these data suggested that each subtype of collagen behaves differently in dependency of stiffness and EF values, the highest fractions being achieved for type V collagen.

## Discussion

We analysed the natural process of left ventricle remodelling in murine mice both *in vivo* and *ex vivo* during early and late periods after MI. While prior studies have shown the importance of morphological modifications of left ventricle chamber^2,3^, the most striking spatio-temporal association of the biomechanical impact of fibrillar collagen on the left ventricle remodeling is to be made. Our results highlight the hitherto unknown important communication between remote and scar tissues as their distinct remodelling patterns and biomechanics specifically change during heart remodelling.

After the ischemic event, the contractile ability of myocardium is drastically reduced as indicated by low EF and FS values. The ischemic regions become stiffer than the remote area and control tissue. Since the mean wall thickness is initially similar with that of control, during systole the infarcted region stretches and become stiff, although diastolic filling may remain unaffected^44^.

We assume that the upregulation of type V collagen in remote and scar hinders the early degradation of *de novo* collagen fibers, thus preserving to a certain extent the tissue integrity and its functionality. Our data are supported by previous findings that type V collagen coated cultures prove to be more resistant against MMP-1, -2, and -3 than those of types I and III coated cultures^30^. Beside its protective role, type V collagen may contribute in early co-assembling heterotypic structures of types I and III collagen molecules^28,29^.

At high pressure overloads, the scar increases its stiffness by 2-fold than that of remote and by approximately 3.5 -fold than that of control. By day 4 post-MI, types I and III collagen associate with compensatory EF and further elevated tissue stiffness. Indeed, during early stages post-MI, the scar stiffness stays elevated as stiff types I and III collagen fibers exceed approximately with 3- and 4.5-fold, respectively those of control. Our findings are in-line with the results revealing the potential association between large accumulations of viscoelastic types I and III collagen fibers in the scar and their stabilizing role on the structural and functional integrity of the injured regions^45–47^. Additionally, type V collagen upregulation may play two critical roles: (*i*) supports the biosynthesis of *de novo* collagen fibrils presumably with resilient biomechanical characteristics^28^ and (*ii*) simultaneously protects the collagen matrix against local enzymatic degradation^30^. The upregulation of soft type III collagen may regulate the remote biomechanics to it’s a compensatory regime.

The second phase of heart remodelling is the fibrotic phase, which lasts for roughly 2–3 weeks. This phase is predominated by the abundant biosynthesis of *de novo* collagen molecules as the provisional matrix turns into mature connective tissue. Mainly, large amounts of *de novo* soft type III collagen and short structural fibers of type V collagen enmesh the cardiac cells, into a reduced compliant remote. Higher expression of stiff type I collagen fibers vs. soft type III and type V collagen accumulated in the scar, seems to associate with increase scar stiffness^19^.

Additionally, it was shown that shortly after MI, the inflammatory chemokines like interleukine-6, partially mediate the increased stiffness of titin in remote myocardium^48^. Titin as the major component of sarcomeres has the ability to modulate the remote tissue response according to Frank-Starling mechanism^49,50^. Four days after MI, the increased stiffness of remote region seems to associate with shortening of sarcomere length and their increased stiffness. Although stress -strain mechanical studies of isolated structures showed significant kPa variations between Z-disks regions and M-band^51^, the e*x vivo* mechanical properties of these meso-scale structures and the mechanics transduced to the myocardial muscle are yet to be demonstrated. The high order magnitude values of sarcomeres stiffness in our study reflects the mechanical and structural contribution of the surrounding tissue, to which sample preparation and the mechanics assessment of specimens need to be considered. If titin is involved in this process is yet to be demonstrated. Fourteen days after MI, stiffness of remote myocardium decreases and compensates the overcontracture of infarcted region by thinning the left ventricular wall. Under high tension, sarcomeres begin to stretch elastically to a maximum mean of 2.7 μm. The elevated amounts of coiled soft type III collagen fibers at 14 days post-MI that surrounds myocytes uncoil along to sarcomeres while limiting further expansions of overstretched sarcomeres. However, the local parallel organization of fibers may lead to the increased stiffness of sarcomeres, as demonstrated in case of collagen fibers alignment on heart function post-MI^35^. In contrast, the remote tissue stiffness decreases. The remote tissue stiffness seems to predominantly be modulated by the newly bio-synthesized soft type III collagen over those of types I and V collagen.

Within this phase, myocytes fill barely any scar region, as collagen fibers surrounds them. Collagen fibers may expends over sizes lager than myocytes dimensions, thereby they may distribute isotropically in scar and remote areas. The morpho-mechanical characteristics of sarcomeres and collagen fibers experienced at high tension, seems to majorly contribute to increasing tensile force in contraction for ejection to occur. At the end of these phase, as the compensatory mechanisms are surpassed the EF and FS decrease.

During the last phase of healing, both EF and FS are reduced. The fibrotic maturated collagen tissue is predominated by *de novo* type I collagen, whereas remote is composed majorly by types I and V collagen. In the healing phase, the reinforced scar tissue become more compliant, which apparently doesn’t associate with increased amounts of stiff type I collagen. Soft type III collagen molecules and type V collagen are found to be intertwingled within the same type I fibrils^29^, thus causing presumably the scar to become less stiff than that in the proliferation phase. The *de novo* stress-tolerant, viscoelastic types I and V collagen scaffolds couple myocytes in the remote, which stabilizes the regular contraction of myocytes and can dissipate evenly the tension to the left ventricle wall. The evenly dissipation of tension is presumably enabled by the thin left ventricle wall, too. However, barely any study shows thinning of left ventricle wall during healing phase and additional studies on the dynamical changes of left ventricle wall thickness post-MI are required in this context. In the healing phase, the accumulation of types I and III collagen seems to associate less with EF. Few studies reveal a possible correlation between scar tissue stiffness and cross-linking degree of collagen fibrils^52^.

Owing to complex remodelling process after MI, we cannot exclude that involvement of other molecular and cellular modifications, which in part goes beyond the scope of our study.

Although, our study develops a novel integrative approach merging from biomechanical, immunohistological, structural and functional data, some limitations have to be drawn. Owing to complex remodelling processes after MI, we cannot exclude the involvement of other molecular, cellular and morphological modifications, which however are beyond of the scope of our study. Correlative models relating spatial distribution of collagen composition during MI are still envisaged, and additional studies are planned to complete the study of the mechanical impact of collagen subtypes and their composition on the heart functionality after MI. Currently, the ethical requirements limit the use of increased experimental animals, thereby hindering a complex association as similar to that obtained in clinical studies. Taken together, present results significantly extend current knowledge in regard to the critical molecular and biomechanical regulators of the infarcted heart. Our associative approach included in these pilot results will constitute a common scenario for future correlative clinical studies. The scientific fundament of our approach is the basis for studying the underlying mechanism of heart damage in patients suffering from myocardial infarction.

## Conclusion

**Figure 7:**
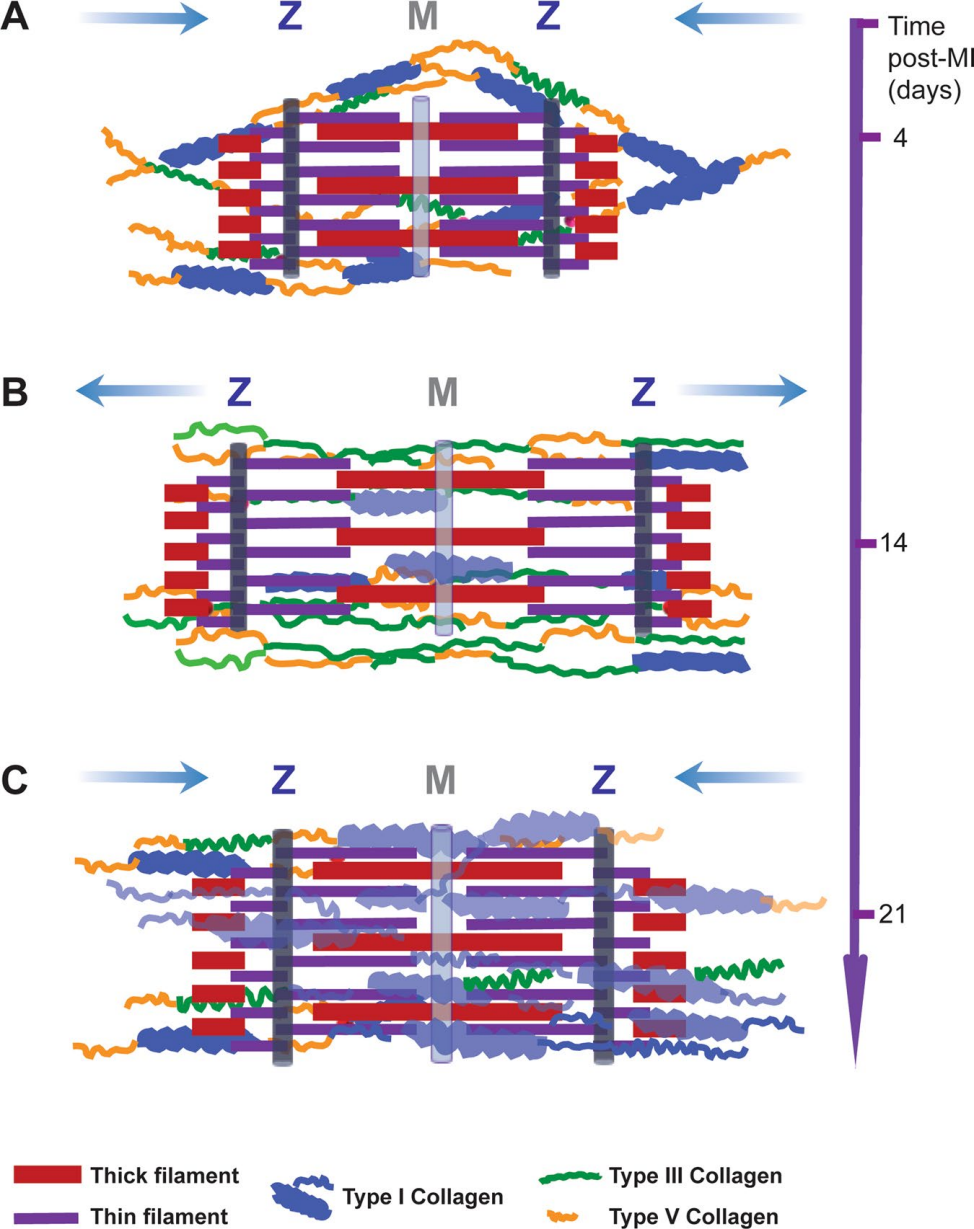
Schematic overview of remote remodelling after MI. (**A**) Early after MI, higher expression of types V and I collagen partially hinders relaxation-contraction of sarcomeres, increasing the remote stiffness and decreasing the sarcomeres’ length. (**B**) Type V collagen supports the biosynthesis of a soft and thin type III collagen fibers. Thus, tissue stiffness decreases while sarcomeres elongate to maintain the heart pump function at increased mechanical overload. (**C**) In the late remodeling phase, the synthesis of type I collagen allows the recovery of tissue compliance and sarcomeres’ length. Blue arrows indicate the reduction - elongation of sarcomeres’ dimensions. The thick blue and grey light lines denote M bands and Z disks of sarcomeres.

Our original data showed that, type V collagen is mandatory to regulate *de novo* collagen fibers’ dimensions of types I and III collagen which is highly relevant for increasing heart tissue stiffness early after MI. The optimum functionality of remote and scar regions counts for the partial contribution of synthesis and accumulation of fractions of *de novo* collagen subtypes whereas cardiomyocytes adapt their contraction ability, thus leading to a temporary biomechanical compensation of the heart (Fig. 7A–C).

Correlative models relating spatial distribution of collagen composition during MI are yet to be envisaged, and additional studies are expected to complete the study on the mechanical impact of collagen subtypes and their composition on the heart functionality after MI. However, our pre-clinical data explain in part the heart failure induced in patient with extended MI. The interdepending dynamics formation and remodeling of adjacent scar and remote tissues are essential events occurring in all organs. We strongly believe that our scientific report brings valuable knowledge and expands the state of the art on tissue formation and remodeling such as remote and scar in their fields.

## References

[1] Benjamin EJ (2017). Heart Disease and Stroke Statistics-2017 Update: A Report From the American Heart Association. Circulation.

[2] Abhilash AS, Baker BM, Trappmann B, Chen CS, Shenoy VB (2014). Remodeling of fibrous extracellular matrices by contractile cells: predictions from discrete fiber network simulations. Biophys J.

[3] Ertl G, Frantz S (2005). Healing after myocardial infarction. Cardiovascular research.

[4] Valderrabano M (2007). Influence of anisotropic conduction properties in the propagation of the cardiac action potential. Prog Biophys Mol Biol.

[5] French BA, Kramer CM (2007). Mechanisms of Post-Infarct Left Ventricular Remodeling. Drug discovery today. Disease mechanisms.

[6] Gupta KB, Ratcliffe MB, Fallert MA, Edmunds LH, Bogen DK (1994). Changes in passive mechanical stiffness of myocardial tissue with aneurysm formation. Circulation.

[7] Fomovsky GM, Holmes JW (2010). Evolution of scar structure, mechanics, and ventricular function after myocardial infarction in the rat. Am J Physiol Heart Circ Physiol.

[8] Richardson WJ, Clarke SA, Quinn TA, Holmes JW (2015). Physiological Implications of Myocardial Scar Structure. Compr Physiol.

[9] Liehn EA (2008). Ccr1 deficiency reduces inflammatory remodelling and preserves left ventricular function after myocardial infarction. J Cell Mol Med.

[10] Liehn EA (2010). A new monocyte chemotactic protein-1/chemokine CC motif ligand-2 competitor limiting neointima formation and myocardial ischemia/reperfusion injury in mice. J Am Coll Cardiol.

[11] Liehn EA, Postea O, Curaj A, Marx N (2011). Repair after myocardial infarction, between fantasy and reality: the role of chemokines. J Am Coll Cardiol.

[12] Moss RL, Fitzsimons DP (2002). Frank-Starling relationship: long on importance, short on mechanism. Circ Res.

[13] Shiels HA, White E (2008). The Frank-Starling mechanism in vertebrate cardiac myocytes. J Exp Biol.

[14] Lakatta, E. G. Length modulation of muscle performance: Frank-Starling law of the heart. *New York, NY**:**Raven Press Publishers*, 1325–1351 (1992).

[15] Rassier DJE (2000). The degree of activation of cardiac muscle depends on muscle length. Arg. Bras. Cardiol..

[16] Gilbert SH, Benson AP, Li P, Holden AV (2007). Regional localisation of left ventricular sheet structure: integration with current models of cardiac fibre, sheet and band structure. Eur J Cardiothorac Surg.

[17] Stevens C, Hunter PJ (2003). Sarcomere length changes in a 3D mathematical model of the pig ventricles. Prog Biophys Mol Biol.

[18] Talman, V. & Ruskoaho, H. Cardiac fibrosis in myocardial infarction-from repair and remodeling to regeneration. *Cell Tissue Res* (2016). 10.1007/s00441-016-2431-9 PMC5010608 27324127

[19] Badenhorst D (2003). Cross-linking influences the impact of quantitative changes in myocardial collagen on cardiac stiffness and remodelling in hypertension in rats. Cardiovasc Res.

[20] Fomovsky GM, Rouillard AD, Holmes JW (2012). Regional mechanics determine collagen fiber structure in healing myocardial infarcts. J Mol Cell Cardiol.

[21] Fomovsky GM, Thomopoulos S, Holmes JW (2010). Contribution of extracellular matrix to the mechanical properties of the heart. J Mol Cell Cardiol.

[22] Rohr S (2009). Myofibroblasts in diseased hearts: new players in cardiac arrhythmias?. Heart Rhythm.

[23] Eghbali M, Weber KT (1990). Collagen and the myocardium: fibrillar structure, biosynthesis and degradation in relation to hypertrophy and its regression. Mol Cell Biochem.

[24] Cleutjens JP, Verluyten MJ, Smiths JF, Daemen MJ (1995). Collagen remodeling after myocardial infarction in the rat heart. The American journal of pathology.

[25] Weber KT, Sun Y, Tyagi SC, Cleutjens JP (1994). Collagen network of the myocardium: function, structural remodeling and regulatory mechanisms. J Mol Cell Cardiol.

[26] Weber KT (1989). Cardiac interstitium in health and disease: the fibrillar collagen network. J Am Coll Cardiol.

[27] Weber KT (1988). Collagen remodeling of the pressure-overloaded, hypertrophied nonhuman primate myocardium. Circ Res.

[28] Wenstrup RJ (2004). Type V collagen controls the initiation of collagen fibril assembly. J Biol Chem.

[29] Asgari M, Latifi N, Heris HK, Vali H, Mongeau L (2017). *In vitro* fibrillogenesis of tropocollagen type III in collagen type I affects its relative fibrillar topology and mechanics. Sci Rep.

[30] Kerkvliet EH, Jansen IC, Schoenmaker T, Beertsen W, Everts V (2003). Collagen type I, III and V differently modulate synthesis and activation of matrix metalloproteinases by cultured rabbit periosteal fibroblasts. Matrix Biol.

[31] Burgess ML (1996). Exercise- and hypertension-induced collagen changes are related to left ventricular function in rat hearts. Am J Physiol.

[32] Linehan KA, Seymour AM, Williams PE (2001). Semiquantitative analysis of collagen types in the hypertrophied left ventricle. J Anat.

[33] Guccione JM (2001). Mechanism underlying mechanical dysfunction in the border zone of left ventricular aneurysm: a finite element model study. Ann Thorac Surg.

[34] Fomovsky GM, Macadangdang JR, Ailawadi G, Holmes JW (2011). Model-based design of mechanical therapies for myocardial infarction. J Cardiovasc Transl Res.

[35] Voorhees AP, Han HC (2014). A model to determine the effect of collagen fiber alignment on heart function post myocardial infarction. Theor Biol Med Model.

[36] Botker HE (2018). Practical guidelines for rigor and reproducibility in preclinical and clinical studies on cardioprotection. Basic Res Cardiol.

[37] Lindsey ML (2018). Guidelines for experimental models of myocardial ischemia and infarction. Am J Physiol Heart Circ Physiol.

[38] Wu Z (2015). Nano-level morphology of scar tissue after myocardial infarction. Discoveries.

[39] Kim Y, Garvin J, Goldstein MK, Meystre SM (2015). Classification of Contextual Use of Left Ventricular Ejection Fraction Assessments. Stud Health Technol Inform.

[40] Pop-Fele L (2016). Advanced modular automated calculation of the morpho-histological parameters in myocardial infarction. Discoveries.

[41] Gomori G (1950). A rapid one-step trichrome stain. Am J Clin Pathol.

[42] Derjaguin B, Muller V, Toporov Y (1975). Effect of contact deformations on the adhesion of particles. J Coll Interface Sci.

[43] Hutter JL, Bechhoefer J (1993). Calibration of Atomic-Force Microscope Tips. Rev Sci Instrum.

[44] Bogen DK, Rabinowitz SA, Needleman A, McMahon TA, Abelmann WH (1980). An analysis of the mechanical disadvantage of myocardial infarction in the canine left ventricle. Circ Res.

[45] Dobaczewski M, de Haan JJ, Frangogiannis NG (2012). The extracellular matrix modulates fibroblast phenotype and function in the infarcted myocardium. J Cardiovasc Transl Res.

[46] Dobaczewski M, Gonzalez-Quesada C, Frangogiannis NG (2010). The extracellular matrix as a modulator of the inflammatory and reparative response following myocardial infarction. J Mol Cell Cardiol.

[47] Jalil JE (1989). Fibrillar collagen and myocardial stiffness in the intact hypertrophied rat left ventricle. Circ Res.

[48] Kotter S (2016). Titin-Based Cardiac Myocyte Stiffening Contributes to Early Adaptive Ventricular Remodeling After Myocardial Infarction. Circ Res.

[49] Li Y, Lang P, Linke WA (2016). Titin stiffness modifies the force-generating region of muscle sarcomeres. Sci Rep.

[50] Lakatta EG (1987). Starling’s law of the heart is explained by an intimate interaction of muscle length and myofilament calcium activation. J Am Coll Cardiol.

[51] Ogneva IV, Lebedev DV, Shenkman BS (2010). Transversal stiffness and Young’s modulus of single fibers from rat soleus muscle probed by atomic force microscopy. Biophys J.

[52] Westermann D (2011). Cardiac inflammation contributes to changes in the extracellular matrix in patients with heart failure and normal ejection fraction. Circ Heart Fail.

